# Association of chronic rhinosinusitis with bronchial asthma and its severity: A protocol for systematic review and meta-analysis

**DOI:** 10.1097/MD.0000000000024772

**Published:** 2021-03-05

**Authors:** Yangwang Pan, Hongrui Zang

**Affiliations:** Department of Otolaryngology Head and Neck Surgery, Beijing Tongren Hospital, Capital Medical University, Key Laboratory of Otolaryngology Head and Neck Surgery, Ministry of Education of China, Beijing, PR China.

**Keywords:** bronchial asthma, chronic rhinosinusitis, meta-analysis, severity

## Abstract

**Background:**

To explore the association of chronic rhinosinusitis (CRS) with bronchial asthma (BA) as well as its severity.

**Methods:**

A comprehensive database search will be performed from PubMed, Embase, Cochrane Library, and Web of science for related literatures. Heterogeneity test will be used to assess each outcome indicator. If heterogeneity statistics *I*^2^ ≥ 50%, the random effects model will be applied; if *I*^2^ < 50%, the fixed effects model will be performed. Sensitivity analysis will be performed in all models. STATA 15.0 software (Stata Corporation, College Station, TX) will be used for statistical analysis. Risk ratio (RR) will be used as the effect size for enumeration data. *P* < .05 is considered statistically significant.

**Conclusion:**

This study will evaluate the association of CRS with the prevalence of BA as well as its severity.

**OSF registration number:**

10.17605/OSF.IO/GCTM9.

## 1 Introduction

Chronic rhinosinusitis (CRS), a common upper airway disease, is characterized by inflammation of the nasal cavity and paranasal sinuses lasting for 12 consecutive weeks.^[1]^ CRS is often accompanied by some nasal symptoms, such as nasal drainage, congestion, and anosmia, which leads to a dramatic decrease in the patient's quality of life.^[2]^ Studies have shown that patients with CRS may have certain comorbidities and the most common one is bronchial asthma (BA).^[3,4]^

BA is a chronic allergic inflammatory disease of the lower airways characterized by reversible airflow obstruction and bronchial hyperresponsiveness. The typical symptoms of asthma are chest tightness, dyspnea, cough, and wheezing.^[5]^ Presently, CRS and BA are considered in the context of the unified airway theory, which describes the upper and lower airways as a single functional unit.^[6,7]^ The relationship between CRS and BA has been widely studied. Studies have shown that rhinosinusitis played a role in triggering asthma attacks, and the symptoms of asthma were observed to relieve after medical or surgical treatment of rhinosinusitis.^[8–10]^ What is more, patients with CRS were more likely to develop asthma.^[11]^ However, a recent report found that there was no correlation between CT-documented sinonasal involvement and asthma severity.^[12]^ In addition, the CT findings of Bresciani et al indicated that CRS was not associated with asthma severity.^[13]^

Although the relationship between CRS and asthma has been demonstrated in a lot of studies, there still exists inconsistency. In addition, little is known about the relationship between CRS and the severity of asthma. Hence, we plan to perform a meta-analysis to figure out the relationship between CRS and asthma as well as its severity.

## 2 Methods

In a study such as meta-analysis, the Institutional Review Board's approval or the informed consent are not required. Our study will be performed documented according to Preferred Reporting Items for Systematic Reviews and Meta-analysis (PRISMA) guidelines.

### 2.1 Protocol registration

Our protocol has been approved by the Open Science Framework (OSF) registries (https://osf.io/gctm9) with the registration number of 10.17605/OSF.IO/GCTM9. The protocol is performed according to the PRISMA guidelines.

### 2.2 Search strategies

**Figure 1:**
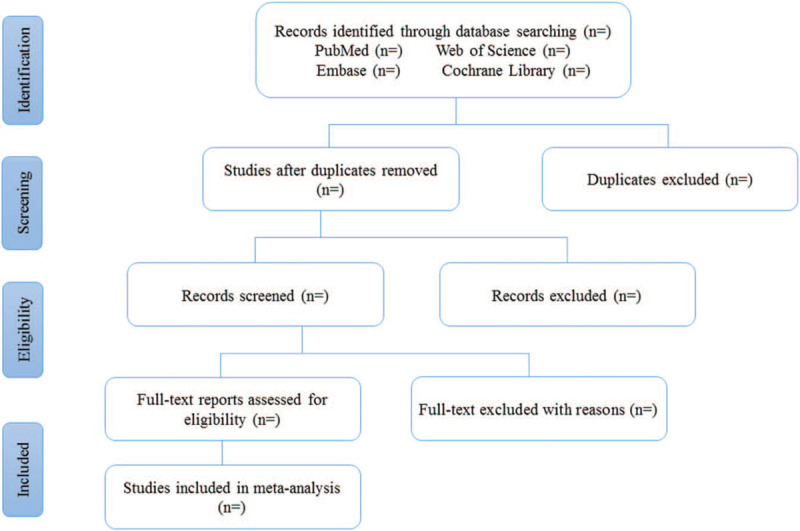
Flow chart of the study.

Databases including PubMed, Embase, Cochrane Library and Web of science will be searched for related literatures. Search strategies will include: ((“Asthma”[Mesh]) OR ((((Asthma [Title/Abstract]) OR (Bronchial Asthma [Title/Abstract])) OR (Asthma, Bronchial [Title/Abstract])) OR (asthmatic [Title/Abstract]))) AND (((((Chronic rhinosinusitis [Title/Abstract]) OR (Sinuses [Title/Abstract])) OR (rhinosinusitis [Title/Abstract])) OR (paranasal sinus disease [Title/Abstract])) OR (CRS [Title/Abstract])). The flow chart of this study is shown in Figure 1.

### 2.3 Eligibility criteria

Inclusion criteria:

1. cohort studies or cross-sectional studies;
2. patients diagnosed with CRS as the study group, and those without CRS as the control group;
3. study outcomes with incidence and severity∗ (mild, moderate and severe) of asthma [The severity of asthma is classified according to the Global Initiative for Asthma (GINA) guidelines in 2006.];
4. English literatures.

Exclusion criteria:

1. meta-analyses, reviews, case reports, conference abstracts, letters, or animal studies;
2. literatures unable to get the full text or extract the data.

### 2.4 Data extraction and quality appraisal

The literatures will be reviewed and the research data will be extracted by 2 researchers (Yangwang Pan and Hongrui Zang) according to the inclusion and exclusion criteria. If there is a conflict, discussion will be performed in 2 parties until the agreement is reached. The contents will be extracted as follows: the first author, year of publication, country, study design, age, etc.

The JBI Grades (Joanna Briggs Institute) will be used to evaluate the quality of cross-sectional studies. It has a total of 20 points, with >14 as low risk of bias and ≤14 as high risk of bias. The Newcastle-Ottawa Scale (NOS) will be used to assess the quality of cohort studies. The total score of this scale is 10 points, with <5 as low or moderate quality and ≥5 as high quality.

### 2.5 Statistical analysis

Heterogeneity test will be used to assess each outcome indicator. If heterogeneity statistics *I*^*2*^ ≥ 50%, the random effects model will be applied; if *I*^*2*^ < 50%, the fixed effects model will be performed. Sensitivity analysis will be performed in all models. STATA 15.0 software (Stata Corporation, College Station, TX) will be used for statistical analysis. Risk ratio (RR) will be used as the effect size for enumeration data. *P* < .05 is considered statistically significant.

## 3 Discussion

Previous studies have reported that CRS is closely associated with the incidence of BA.^[2,14,15]^ Chung et al demonstrated that the odds ratio of patients with CRS was significantly higher than those without CRS. And CRS had an increased prevalence of various comorbidities, where asthma ranked first.^[14]^ Similarly, Alt et al discovered that the prevalence of asthma in patients with CRS was 26% compared to controls at 7.5%.^[2]^ What is more, according to Ostovar et al, 57.3% of subjects with asthma reported having CRS, which indicated that there was an association between CRS and asthma.^[15]^ For the relationship between CRS and the severity of asthma, Matsuno et al only demonstrated a significant correlation between the asthma severity and sinus morphologic abnormalities in patients with and without sinusitis, which cannot prove sinus disease could directly affect the intensity of asthma.^[16]^ Similarly, the CT findings of Bresciani study suggested that CRS was not related to the severity of asthma.^[13]^ However, several studies have confirmed the relationship between CRS and the severity of asthma.^[17–19]^ In Aazami study, 14% of asthmatics with CRS were in the severe stage, while only 4.2% of patients with simple asthma who were in this stage. This difference was statistically significant.^[17]^ The asthma severity in this meta-analysis was assessed retrospectively from the level of treatment required to control symptoms and exacerbations,^[20]^ while the classification in Aazami study was based on clinical signs and FEV1. In addition, some studies also have found that asthma severity may have a significant correlation with the presentation of CRS.^[18,19]^ According to the research by Tay et al,^[19]^ the prevalence of asthmatics with CRS ranges from 22% to 42%,^[21,22]^ and it rose to 45% for severe asthmatics.^[23]^ The results demonstrated a bidirectional relationship between CRS and the severity of asthma, which may support the unified airway theory in some way. Due to the inconsistency, we will perform this meta-analysis to figure out whether there is association between CRS and asthma severity. To our knowledge, few studies especially systematic reviews and meta-analyses focused on the association of CRS with the severity of asthma. Our study may help increase the awareness of clinicians to provide optimal patient care and improve the prognosis of CRS and asthma.

## Author contributions

**Conceptualization:** Yangwang Pan, Hongrui Zang.

**Writing – original draft:** Yangwang Pan.

**Writing – review & editing:** Hongrui Zang.

## References

[1] Fokkens WJ Lund VJ Mullol J. European position paper on rhinosinusitis and nasal polyps 2012. Rhinol Suppl 2012;23: 3 p preceding table of contents 1–298. 22764607

[2] Alt JA Thomas AJ Curtin K. Mortality risk in patients with chronic rhinosinusitis and its association to asthma. Int Forum Allergy Rhinol 2017;7:591–9. 28272838 10.1002/alr.21931 PMC5462840

[3] Joe SA Thakkar K. Chronic rhinosinusitis and asthma. Otolaryngol Clin North Am 2008;41:297–309. 18328369 10.1016/j.otc.2007.11.001

[4] Dixon AE. Rhinosinusitis and asthma: the missing link. Curr Opin Pulm Med 2009;15:19–24. 19077701 10.1097/MCP.0b013e32831da87e PMC2774711

[5] Massoth L Anderson C McKinney KA. Asthma and chronic rhinosinusitis: diagnosis and medical management. Med Sci (Basel) 2019;7:53. 10.3390/medsci7040053 PMC6524348 30934800

[6] Licari A Castagnoli R Denicolò CF. The nose and the lung: united airway disease? Front Pediatr 2017;5:44. 28316969 10.3389/fped.2017.00044 PMC5334318

[7] Stachler RJ. Comorbidities of asthma and the unified airway. Int Forum Allergy Rhinol 2015;5: Suppl 1: S17–22. 26335831 10.1002/alr.21615

[8] Ragab S Scadding GK Lund VJ. Treatment of chronic rhinosinusitis and its effects on asthma. Eur Respir J 2006;28:68–74. 16510462 10.1183/09031936.06.00043305

[9] Smart BA. Is rhinosinusitis a cause of asthma? Clin Rev Allergy Immunol 2006;30:153–64. 16785587 10.1385/CRIAI:30:3:153

[10] Kalogjera L Vagić D Baudoin T. Effect of endosinusal treatment on cellular markers in mild and moderate asthmatics. Acta Otolaryngol 2003;123:310–3. 12701765 10.1080/00016480310001178

[11] Bachert C Vignola AM Gevaert P. Allergic rhinitis, rhinosinusitis, and asthma: one airway disease. Immunol Allergy Clin North Am 2004;24:19–43. 15062425 10.1016/S0889-8561(03)00104-8

[12] Raherison C Montaudon M Stoll D. How should nasal symptoms be investigated in asthma? A comparison of radiologic and endoscopic findings. Allergy 2004;59:821–6. 15230813 10.1111/j.1398-9995.2004.00487.x

[13] Bresciani M Paradis L Des Roches A. Rhinosinusitis in severe asthma. J Allergy Clin Immunol 2001;107:73–80. 11149994 10.1067/mai.2001.111593

[14] Chung SD Chen PY Lin HC. Comorbidity profile of chronic rhinosinusitis: a population-based study. Laryngoscope 2014;124:1536–41. 24395611 10.1002/lary.24581

[15] Ostovar A Fokkens WJ Pordel S. The prevalence of asthma in adult population of southwestern Iran and its association with chronic rhinosinusitis: a GA2LEN study. Clin Transl Allergy 2019;9:43. 31497279 10.1186/s13601-019-0283-6 PMC6717339

[16] Matsuno O Ono E Takenaka R. Asthma and sinusitis: association and implication. Int Arch Allergy Immunol 2008;147:52–8. 18451648 10.1159/000128659

[17] Aazami A Sharghi A Ghofrani M. Rhinosinusitis predispose asthmatic patients to severe bronchial asthma. Iran J Allergy Asthma Immunol 2009;8:199–203. 20404390

[18] Lin DC Chandra RK Tan BK. Association between severity of asthma and degree of chronic rhinosinusitis. Am J Rhinol Allergy 2011;25:205–8. 21819754 10.2500/ajra.2011.25.3613 PMC3390198

[19] Tay TR Hew M. Comorbid “treatable traits” in difficult asthma: current evidence and clinical evaluation. Allergy 2018;73:1369–82. 29178130 10.1111/all.13370

[20] Gina G. Global strategy for asthma management and prevention: GINA executive summary. Eur Respir J 2008;31:143–78. 18166595 10.1183/09031936.00138707

[21] Ek A Middelveld RJ Bertilsson H. Chronic rhinosinusitis in asthma is a negative predictor of quality of life: results from the Swedish GA (2) LEN survey. Allergy 2013;68:1314–21. 24107218 10.1111/all.12222

[22] Liou A Grubb JR Schechtman KB. Causative and contributive factors to asthma severity and patterns of medication use in patients seeking specialized asthma care. Chest 2003;124:1781–8. 14605049 10.1378/chest.124.5.1781

[23] Moore WC Meyers DA Wenzel SE. National heart, lung, and blood institute's severe asthma research program. identification of asthma phenotypes using cluster analysis in the severe asthma research program. Am J Respir Crit Care Med 2010;181:315–23. 19892860 10.1164/rccm.200906-0896OC PMC2822971

